# Gender-related alterations in plasma adrenomedullin level and its correlation with body weight gain

**DOI:** 10.1530/EC-14-0131

**Published:** 2015-02-02

**Authors:** Sayaka Kawano, Yukiko Kawagoe, Kenji Kuwasako, Satoshi Shimamoto, Koji Igarashi, Mariko Tokashiki, Kazuo Kitamura, Johji Kato

**Affiliations:** 1 Frontier Science Research Center, University of Miyazaki, 5200 Kihara, Kiyotake, Miyazaki, 889-1692, Japan; 2 Circulatory and Body Fluid Regulation, Department of Internal Medicine, Faculty of Medicine, University of Miyazaki, 5200 Kihara, Kiyotake, Miyazaki, 889-1692, Japan; 3 AIA Research Group, Bioscience Division, Reagent Development Department, TOSOH Corporation, Kanagawa, 252-1123, Japan

**Keywords:** adrenomedullin, plasma level, gender difference, body weight gain

## Abstract

Plasma levels of adrenomedullin (AM), a bioactive peptide produced in adipose tissue, have been shown to be higher in obese patients than in non-obese patients, but little is known about gender differences in plasma AM levels. The aims of this study were to clarify gender-related alterations in plasma AM levels and to examine the body weight (BW) gain–plasma AM relationship in the general population. We measured plasma AM levels of 346 local residents (62.0±8.9 years, mean±s.d.) in the Kiyotake area, Japan, who underwent a regular health check-up, by a specific fluorescence immunoassay. Plasma AM levels in the female residents were lower than that in the males, and multiple regression analysis revealed a possible gender difference in plasma AM. The AM levels were significantly correlated with BMI or waist circumference in women, but such a relationship was not seen in men. When the subjects were divided into two groups by results of a questionnaire about BW gain of 10 kg or more since the age of 20 years, the plasma AM level of women with BW gain ≧10 kg was significantly higher than that in those without BW gain, although no difference was noted between the men with and without BW gain. In conclusion, possible gender differences were noted in the plasma AM levels and in the BW gain–plasma AM relationship in the general population. The plasma AM levels in the female residents without BW gain seem partly attributable to the lower AM of women.

## Introduction

Adrenomedullin (AM) is a potent vasodilator peptide first isolated from human pheochromocytoma tissue, although this peptide has also been shown to be present in a wide range of human tissues or organs: the adrenal medulla, heart, blood vessels, kidneys, and adipose tissues (1, 2, 3). A number of functional analysis studies have been carried out since its discovery, revealing that AM has pleiotropic actions, including blood pressure-lowering effects, natriuresis, cardiovascular protection, and alleviation of insulin resistance (4, 5, 6, 7). On the other hand, AM was shown to circulate in human blood, and its plasma levels are elevated in diseases such as hypertension, heart failure, and sepsis (2, 8). Plasma AM levels have also been found to be higher in patients with obesity than in control subjects, suggesting the active production and secretion of AM from human adipose tissue (6, 9). Consistent with this, Nambu *et al*. (10) reported that mice fed a high-fat diet showed augmented AM expression in the fat tissues and elevated plasma levels concomitant with increased body weight (BW). As inflammatory cytokines, including tumor necrosis factor alpha (TNFα) and interleukin 1 beta (IL1β), were reported to increase AM production, low-grade inflammation in fat tissues associated with obesity is assumed to be involved in the increased expression of AM (6, 8, 11). When evaluating plasma levels of endogenous bioactive substances, we sometimes need to take gender-related alterations into account. For instance, plasma levels of brain natriuretic peptide (BNP), a bioactive peptide exerting vasodilatory and blood pressure-lowering effects, are higher in women than in men (12). Another example is the renin–angiotensin–aldosterone system: estrogen, a sex steroid hormone, has been shown to modulate this system in various manners (13). Meanwhile, as indicated by a search of the literature, little is known about gender-related alterations in plasma AM levels or AM production. The aims of this study are thus to clarify whether there are gender-related alterations in plasma AM level and to determine the relationship between BW gain and plasma AM levels in human subjects by examining local residents in a Japanese community using a newly developed, automated, AM-specific fluorescence immunoassay. In addition to AM, we measured plasma levels of BNP and an N-terminal fragment of the BNP precursor (NT-proBNP) in order to compare gender- or BW gain-related alterations in AM with those of the BNP peptides.

## Materials and methods

### Study subjects and protocol

Local residents of the Kiyotake area, Miyazaki, Japan, who underwent an annual regular health check-up as part of a specific health program of the Japanese government from 2008 to 2013, were randomly selected for this study (172 males and 174 females; 62.0±8.9 years, mean±s.d.). Upon visiting the community center of Kiyotake Town, the medical history of the residents, which included the questionnaire of whether or not they have had BW gain of 10 kg or more since 20 years of age, was taken by nurses, and blood pressure was measured with an oscillometric automatic device (BP-103iII, Colin, Nagoya, Japan) in a sitting position. The history taken was confirmed by physicians, who then carried out physical examination including auscultation. Exclusion criteria were as follows: first, residents with medical history, symptoms, or signs indicative of any heart disease were excluded because the plasma levels of AM, BNP, and NT-proBNP were shown to be elevated in patients with ischemic heart disease or heart failure (8, 14). Second, renal function is an important determinant of those peptide levels (14, 15), therefore we excluded residents with estimated glomerular filtration rate (eGFR) of 30 ml/min per 1.73 m^2^ or lower. eGFR was calculated with the following formula of the Japanese Society of Nephrology (16): 194×serum creatinine^−^^1.094^×age^−^^0.287^ ml/min per 1.73 m^2^, further multiplied by 0.739 for women. Lastly, because elevated blood glucose was shown to increase plasma AM levels (17, 18), we excluded residents whose HbA1c was 6.5% or higher, so as not to select those with uncontrolled diabetes mellitus.

This study was approved by the Review Committee for Cooperative and Commissioned Research and the Ethics Committee of the University of Miyazaki Faculty of Medicine. All subjects examined gave their written informed consent before participating in this study.

### Measurements of bioactive peptides in plasma

To measure the plasma levels of AM, BNP and NT-proBNP after overnight fasting, blood from an antecubital vein was collected into tubes with 1.0 mg/ml EDTA-2Na and 500 kallikrein inhibitory units (KIU)/ml of aprotinin. Plasma was obtained by centrifugation at 1710 ***g*** for 10 min at 4 °C and stored at −30 °C until the assay. Plasma levels of AM were measured by a specific fluorescence immunoassay (Tosoh Corporation, Tokyo, Japan) with two independent antibodies: one binds to the ringed structure and the other to the middle region between the ring and C-terminal portions of the peptide, as previously described (9, 19). The AM assay reagent prepared by an immediate freeze-dry procedure was composed of in-house magnetic beads coated with the anti-ringed structure antibody and alkaline phosphatase-labeled anti-C-terminal portion antibody. This assay reagent can be used with a commercially available, automated immunoassay analyzer (AIA-System, Tosoh Corporation). The limits of detection and quantitation of this assay were determined to be 0.133 and 0.085 pmol/l, respectively, according to the Clinical and Laboratory Standards Institute (CLSI) protocols. Intra- and inter-assay coefficients of variation of this assay were 1.8% (*n*=10) and 5.1% (*n*=11) respectively. BNP and NT-proBNP levels were determined by chemiluminescent immunoassay (Shionogi & Co. Ltd, Osaka, Japan) and electro-chemiluminescence assay (Roche Diagnostics) respectively (20, 21).

### Statistical analysis

All the data were analyzed using IBM SPSS Software, version 22.0 (IBM, Armonk, NY, USA). Two groups were compared by the unpaired *t*-test or *χ* ^2^ test, while multiple comparisons were made by ANOVA followed by Scheffe's test. Simple regression analysis was used to examine the relationships between plasma levels of the peptides and the other parameters, and these relationships were further tested by Spearman's rank correlation coefficient. A multiple linear regression analysis with a stepwise method was used to extract factors significantly associated with the plasma AM levels. All data are expressed as the means±s.d. and *P*<0.05 was considered to be statistically significant.

## Results

The basal profiles and peptide measurements of the residents examined in this study are given in Table 1. The plasma level of AM in the female residents was significantly (*P*<0.01) lower than that in the males, while in contrast, the BNP and NT-proBNP levels were slightly higher in women than in men. When men and women were analyzed together by simple regression analysis, the AM levels were significantly correlated with BMI (*r*=0.153, *P*<0.01) and waist circumference (WC; *r*=0.132, *P*<0.05). As there were substantial differences in the basal profiles between the two genders (Table 1), we further analyzed the data to examine whether gender is independently associated with the plasma AM levels by multiple regression analysis with a stepwise method. The parameters included as explanatory covariates in this analysis were the BMI, mean blood pressure, and fasting blood glucose level, because these parameters, in addition to prevalence of hypertension, significantly differed between men and women (Table 1 and Supplementary Table 1, see section on supplementary data given at the end of this article). Also those included were age and eGFR, which have been reported to be the factors influencing plasma AM levels (15, 22). As given in Table 2, although marginally significant (*P*=0.043), gender was extracted as an independent determinant of the plasma AM levels, in addition to BMI and eGFR, in the study subjects.

**Figure 1:**
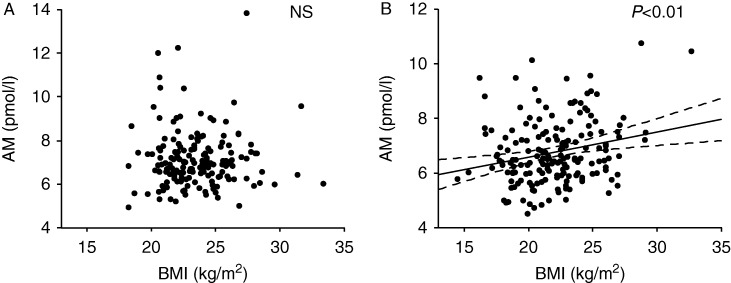
Relationships between BMI and plasma AM levels in the male (A) and female (B) residents. The regression line and the 95% confidence limits are shown by solid and broken lines respectively. NS, not significant.

We then analyzed the data of the male and female residents separately. The relationships between plasma levels of the peptides and the BMI or WC are given in Table 3 as Pearson's correlation coefficients (*r*). The plasma levels of AM were found to be correlated with the BMI and WC in women, but such a relationship was not detected in men, as also shown in Fig. 1A and B. These results were confirmed by Spearman's rank correlation coefficient, which showed significant relationships between the plasma AM and BMI or WC in women but not in men (data not shown). In contrast to AM, as given in Table 3, inverse correlations were found between the plasma levels of BNP or NT-proBNP and the BMI or WC in women.

Basal profiles of the study subjects with or without BW gain of 10 kg or more since 20 years old are given in Table 4. Compared with the residents without BW gain, significantly higher (*P*<0.01) values were noted in the BW, BMI, and WC in those with BW gain, although there were no significant differences in the other clinical parameters, including age, blood pressure, blood glucose, HbA1c, and renal function, between the two groups. Prevalence of hypertension in the male residents was higher than that in the females (Supplementary Table 1), but when compared within the same gender, no differences were noted for hypertension, dyslipidemia, or diabetes mellitus (Supplementary Table 2, see section on supplementary data given at the end of this article). As given in Table 5, when comparing the residents with and without BW gain, we failed to detect a difference in the plasma AM levels of the males, but found a significantly higher level of plasma AM in the female residents with BW gain (*P*<0.01). In comparison between the two genders without BW gain, the plasma AM level in women was significantly lower than that in men (*P*<0.01). The plasma levels of BNP and NT-proBNP in the male and female residents with BW gain were slightly lower than that in those without, but the differences did not reach statistically significant levels. As expected, the plasma levels of two BNP peptides were slightly, but not significantly, higher in the female residents than that in the males, irrespective of BW gain.

## Discussion

Plasma levels of AM, a bioactive peptide with pleiotropic actions, are increased in various human diseases including hypertension, heart failure, and obesity (3, 8), but little is known about gender-related differences in plasma AM. According to an animal study, BW gain via a high-fat diet resulted in augmented AM expression in adipose tissue with concomitant elevation of plasma AM levels in rats (10), but BW gain-induced elevation of plasma AM level has not been proven in humans yet. Examining the general population in this study, we revealed that i) plasma AM levels in women might be lower than that in men; ii) AM levels in women are associated with BW gain and a possible gender-related alteration is noted in the plasma AM–BW gain relationship; and iii) the lower AM levels in women are likely due to those without BW gain.

An important issue that arises in this study is the mechanism for the closer relationship between BW gain and the plasma AM levels in the female residents compared with that in the males. Currently, there is no clear explanation for this, but we can discuss some possibilities based on previous reports. It has been shown that the factors affecting plasma AM levels in humans without overt cardiovascular or renal diseases are age, BMI, blood pressure, and renal function (6, 22). In this study, no differences were noted in those parameters between the subjects with or without BW gain in both genders, except for BW, BMI, and WC.

As fat tissue appears to be an organ contributing to AM circulation in human blood (10, 23), it is possible that a gender difference in BW gain-induced production of AM in the adipose tissue accounts for the present phenomenon. According to a report by Paulmyer-Lacroix *et al*. (23), expression of AM is augmented in the omental adipose tissue of obese women compared with that in the non-obese. In this study, the BW gain-induced elevation of plasma AM may have resulted from increased expression of AM in the visceral fat of the female residents. This is unlikely to be the case in male residents because there was no difference in the plasma AM levels in those with or without BW gain, despite the substantial differences in BW, BMI, and WC; however, there have been no reported studies comparing AM expression in adipose tissue between non-obese and obese men.

Low-grade inflammation in adipose tissues associated with obesity seems involved in the mechanism of the increased plasma AM level in obese subjects, because the AM production is up-regulated by inflammatory cytokines such as TNFα or IL1β (6, 8, 9, 11). It was reported that body fat distribution differs from between two genders: ratios of the visceral fat to the subcutaneous or lower body fat mass were higher in men than in women (24). According to an epidemiological study by Pou *et al*. (25), increased volumes of the visceral and subcutaneous fat were associated with elevation of inflammatory markers, while the former was more closely related to these markers than the latter. In this context, the intimate relationship between the plasma AM and BW gain in women of this study is somehow contradictory. Although there are no data available about menopausal state in this study, it seems unlikely that sex steroids are involved in the gender difference in BW gain-induced AM production: neither testosterone nor estradiol has much effect on AM production (26). Clearly, further studies are necessary to clarify the mechanism behind the gender difference in BW gain-induced alteration in plasma AM levels.

BNP has natriuretic and vasodilatory effects, exerting cardiovascular protective actions, and plasma levels of BNP are elevated in patients with hypertension and heart failure, as are those of AM (14). The increased BNP levels are thought to be a mechanism counteracting blood pressure elevation and excess body fluid retention in patients with hypertension or heart failure (12, 14). In contrast to these phenomena, plasma BNP level has been shown to be decreased in obesity, where reduced BNP action is assumed to be involved in BW gain-induced elevation of blood pressure (27). Indeed, higher BNP or NT-proBNP levels were found to be associated with favorable adipose tissue distribution by a population-based study (28). Chainani-Wu *et al*. (29) reported increased plasma BNP levels in obese patients with coronary heart disease (CHD) or high risk of CHD following comprehensive life style modification, suggesting that the BNP elevation associated with BW reduction does not necessarily indicate deterioration of heart disease.

In this study, consistent with the notions discussed above, plasma levels of BNP and NT-proBNP in the subjects with BW gain were slightly lower than in those without BW gain in both genders. In addition, plasma levels of these peptides were inversely correlated with BMI or WC in the female subjects; however, the differences between the residents with or without BW gain were not statistically significant in both genders. This study also showed the higher plasma BNP and NT-proBNP levels in women than in men, a finding accordant with the previous notions (12), while those gender difference were less clear as compared with plasma AM. Thus, the present results suggest that both gender- and BW gain-related alterations in plasma levels of the peptides are clearer in AM than in BNP.

Next, we need to discuss the biological or clinical significance of the present findings. AM has been shown to exert a wide range of biological actions including blood pressure lowering, cardiovascular protection, and alleviation of insulin resistance (3, 5). As mentioned above, in the case of BNP, BW gain-related reduction in plasma BNP levels is assumed to be involved in obesity-induced elevation of blood pressure (27). In contrast to this, we speculate, based on the AM actions, that BW gain-induced increase in AM level in the female subjects is a counter-regulatory mechanism against obesity-related disorders such as insulin resistance and hypertension.

Lastly, there are limitations we need to mention in this study. First, a lack of statistical power may need to be taken into account, because we examined a relatively small number of subjects with BW gain data based on the simple questionnaire. For example, differences in the plasma BNP or NT-proBNP levels between two genders or between those with and without BW gain were statistical insignificant. Meanwhile, a significant finding of this study is that the gender-related alterations were clearly seen in the plasma levels of AM despite insignificant differences in those of the BNP peptides. Second, we have been unable to completely exclude residents with inflammatory, respiratory, or liver diseases, which had possibly affected the AM measurement from the study subjects (3, 6, 8). In our health check-up, when physicians notice possibilities of these diseases in history taking or physical examination, they are supposed to describe it on the medical files; but there were no reports about such a disease. Thirdly, this study lacks parameters or clinical tests, with which we could seek further the relationships between AM levels and low-grade inflammation associated with obesity or alterations of body fat distribution (6, 11), such as C-reactive protein and magnetic resonance imaging, and these points need to be clarified in future studies.

In summary, there appear to be gender-related differences in the plasma AM levels and in the BW gain–plasma AM relationship in the general population. The AM levels in the female residents without BW gain during the adolescent period were partly attributed to the lower plasma AM of women.

## Supplementary data

This is linked to the online version of the paper at http://dx.doi.org/10.1530/EC-14-0131.

## Figures and Tables

**Table 1:** Basal profiles and plasma levels of the peptides of the male and female residents examined in this study. Means±s.d.

	**Men**	**Women**
*n*	172	174
Age (years)	62.1±9.1	61.9±8.6
BMI (kg/m^2^)	23.5±2.5	22.0±3.0*
Waist circumference (cm)	85.1±7.1	82.0±9.1*
Mean blood pressure (mmHg)	96±12	90±12*
Fasting blood glucose (mg/dl)	96±13	92±9*
HbA1c (%)	5.5±0.3	5.5±0.3
eGFR (ml/min per 1.73 m^2^)	74±14	76±14
AM (pmol/l)	7.14±1.29	6.77±1.18*
BNP (pg/ml)	19.1±23.4	21.7±15.5
NT-proBNP (pg/ml)	60.5±101.8	68.1±48.2

eGFR, estimated glomerular filtration rate; AM, adrenomedullin; BNP, brain natriuretic peptide; NT-proBNP, N-terminal proBNP. *P<0.01 vs male residents.

**Table 2:** Identification of significant factors for plasma AM levels by multiple regression analysis with a stepwise method.

**Independent variables**	***β***	***P***
BMI	0.129	0.022
eGFR	−0.119	0.028
Gender (male=1 and female=2)	−0.114	0.043

eGFR, estimated glomerular filtration rate.

**Table 3:** Correlation coefficients (*r*) of simple regression analysis for relationships between BMI or WC and AM, BNP, or NT-proBNP.

***r***	**Men**		**Women**	
BMI	WC	BMI	WC
AM	0.002	0.008	0.231^†^	0.195*
BNP	−0.009	0.027	−0.151*	−0.156*
NT-proBNP	0.012	0.057	−0.155*	−0.108

AM, adrenomedullin; BNP, brain natriuretic peptide; NT-proBNP, N-terminal proBNP; WC, waist circumference. *P<0.05 and ^†^ *P*<0.01, Pearson's correlation.

**Table 4:** Basal profiles of the male and female residents with or without BW gain of 10 kg or more. Means±s.d.

**BW gain ≧10 kg**	**Men**		**Women**	
(−)	(+)	(−)	(+)
*n*	112	60	122	52
Age (years)	62.7±9.4	61.1±8.4	61.3±8.8	63.2±8.1
BW (kg)	60.7±7.3	69.7±8.6*	49.1±5.9	56.8±7.0*
BMI (kg/m^2^)	22.4±1.9	25.5±2.5*	21.0±2.5	24.4±2.6*
Waist circumference (cm)	82.3±5.6	90.4±6.6*	79.3±8.0	88.4±8.0*
Mean blood pressure (mmHg)	95±11	98±13	89±12	91±11
Fasting blood glucose (mg/dl)	96±15	97±11	91±8	94±10
HbA1c (%)	5.4±0.3	5.5±0.3	5.4±0.2	5.5±0.3
eGFR (ml/min per 1.73 m^2^)	73±14	75±14	76±13	76±15

eGFR, estimated glomerular filtration rate. *P<0.01 vs without body weight (BW) gain ≧10 kg.

**Table 5:** Plasma levels of AM, BNP, and NT-proBNP of the male and female residents with or without BW gain of 10 kg or more. Means±s.d.

**BW gain ≧10 kg**	**Men**		**Women**	
(−)	(+)	(−)	(+)
AM (pmol/l)	7.11±1.22	7.20±1.42	6.53±1.02^†^	7.34±1.33*
BNP (pg/ml)	19.6±26.8	18.2±15.5	22.8±15.9	19.2±14.4
NT-proBNP (pg/ml)	64.7±120	52.6±51.8	70.4±47.8	62.6±49.4

AM, adrenomedullin; BNP, brain natriuretic peptide; NT-proBNP, N-terminal proBNP. *P<0.01 vs without body weight (BW) gain in the identical gender and ^†^ *P*<0.01 vs men without BW gain.
